# The In Vitro and In Vivo Analysis of Mammalian Tumour Viruses

**DOI:** 10.1038/bjc.1959.33

**Published:** 1959-06

**Authors:** M. Fogel, L. Sachs


					
266

THE IN VITRO AND IN VIVO ANALYSIS OF MAMMALIAN

TUMOUR VIRUSES

THE HAEMAGGLUTINATING SYSTEM OF THE POLYOMA VIRUS

M. FOGEL AND L. SACHS

From the Department of Experimental Biology, The Isaac Wolfson Building,

Weizmann Institute of Science, Rehovoth, Israel

Received for publication April 23, 1959

SINCE the classical work of Ellerman and Bang (1908) on chicken leukaemia
and of Rous (1911) on chicken sarcoma first demonstrated the viral origin of
certain tumours, such studies have been extended to various species including
mammals (Rous, 1935; Oberling and Guerin, 1954; Gross, 1958). It was shown
in our previous investigations (Sachs, Fogel and Winocour, 1959a; Sachs et al.,
1959b, Winocour and Sachs, 1959), that basic questions relating to the role of
viruses in the origin of mammalian tumours can best be answered by combining
an in vivo with an in vitro analysis, and that by using such a combined approach
it is possible to establish particularly favourable experimental systems. One of
the viruses used in these studies was the polyoma virus (Eddy et al., 1958a)
originally isolated from AK mice with lymphatic leukaemia (Gross, 1958; Stewart,
Eddy and Borgese, 1958). This virus can agglutinate red blood cells from various
species at refrigerator (4? C.) temperature (Eddy et al., 1958a; Sachs, Fogel and
Winocour, 1959a), and it was shown that this ability for haemagglutination can
be used both for an in vitro virus identification, and to determine various aspects
of virus behaviour (Sachs, Fogel and Winocour, 1959a; Sachs et al., 1959b).

In order to establish more fully the characteristics of this virus, and to com-
pare these with non-tumour forming viruses, a further investigation was carried
out on its haemagglutinating system. The present studies are therefore concerned
with the properties of the haemagglutinating particles, the formation of haemag-
glutination inhibition antibodies after virus inoculation in relation to tumour
formation, and the existence of such antibodies in AKR mice.

MATERIALS AND METHODS

Animals

The Swiss mice used for the tissue cultures and virus inoculations into mice,
are derived from a line that has been brother x sister mated for at least 20
generations. The AKR mice are derived from breeding pairs originally obtained
from the Roscoe B. Jackson Memorial Laboratory, Bar Harbor, and the golden
hamsters from a randomly bred colony originating in this country.
Virus

The polyoma virus stocks used for the present experiments were from lines
IL 1-6; and from a line isolated (Sachs et al., 1959b) from leukaemic C3HGr

HAEMAGGLUTINATING SYSTEM OF POLYOMA VIRUS

mice kindly supplied by Dr. Ludwick Gross and inoculated by him with his strain A
leukaemic virus (Gross, 1957). The IL lines, which were obtained after culturing
of tumour cells (Sachs et al., 1959b), consist of isolations 1 and 4 from mouse
parotid tumours. 2 and 5 from mouse mammary adenomas, 3 from a mouse
mammary adenocarcinoma, and 6 from a rat kidney sarcoma. The tumours used
for these isolations were induced with virus derived from strain 3919 (Steward,
Eddy and Borgese, 1958) kindly supplied by Dr. Sarah E. Stewart and Dr. Bernice
E. Eddy. All virus stocks were made and used in a medium consisting of 0.5
per cent lactalbumin hydrolysate in Earle's saline and 20 per cent horse serum.

Haemagglutination

Haemagglutination tests were routinely made with guinea-pig red blood cells,
using 0.15 c.c. of a 1 per cent suspension in saline added to 0 5 c.c. of each dilution
of virus. The saline was adjusted with NaOH to pH 6.8-7.2. Virus dilutions were
made in saline. Blood was taken from the heart in Alsever's solution, and used on
the same day. Sedimentation of the controls was found to be inadequate, if the
red blood cells were kept more than 4 days after collection. In order to standardise
the 1 per cent suspension, 1 c.c. of the cell suspension was mixed with 4 c.c. of
double distilled water and after complete haemolysis the concentration of haemo-
globin measured in a Coleman Junior spectrophotometer at a wavelength of
545 m/u. An optical density of 0.26 was taken to be equivalent to a 1 per cent
suspension of red blood cells. The pattern method of agglutination (Salk, 1944)
was used, and the tests read after 3 hours or overnight at 4? C. Since the agglu-
tination is destroyed at room temperature, the tests were read immediately after
the tubes were taken from the refrigerator.

Haemagglutination inhibition

Inhibition tests were made using 0.25 c.c. of antiserum, previously heated at
56? C. for 30 minutes, and 0.25 c.c. of virus suspension containing 16 haemag-
glutinating units of virus. Complete inhibition was used as the end point. Before
adding the red blood cells, the virus antiserum mixture was incubated for 30
minutes at 37? C. Serum from normal Swiss mice gave inhibition titres up to
1: 40.

EXPERIMENTAL

The haemagglutination spectrum

The polyoma has a specific haemagglutination spectrum when tested with red
blood cells from various species (Sachs, Fogel and Winocour, 1959a). The results
with two lines of this virus, and a comparison with the spectrum of some other
haemagglutinating viruses (data from Smadel (1948) and Burnet and Stone (1946))
are shown in Table I. The two lines of polyoma used were line IL-3, and the line
isolated from a C3HGr leukaemic mouse (Sachs et al., 1959b). Although we have
so far found no haemagglutination with cells from our hamsters, this species has
been reported as positive by Eddy et al. (1958a). This may be due to variation
within the species or the existence of virus mutants. The most rapid reading was
given by cells from the frog Hyla arborea, where there was complete sedimentation
after 30 minutes at 4? C. in contrast to the usual 3 hours with cells from other
species. The cells from this frog also gave the highest titre (Sachs, Fogel and

267

268                     M. FOGEL AND L. SACHS

TABLE I.-Haemagglutination Spectrum of Polyoma Compared to some other

Haemagglutinating Viruses

Red blood cells

Pneu-
Virus   Influ-    Influ-   New-              monia
Poly-   from    enza     enza     castle            virus of
oma    Gross     A        B      disease  Vaccinia   mice

Gainea-pig.    .     . +  .  +   .   +             *   + ?      +        -        -
Itat    .   .    .     .     -   .    ?       i    .   +        ?

Mouse     .    .      +      +        ?                +        ?     i          +

Hamster   .    .    .  -* .   -  . N.D. . N.D. . N.D. . N.D. .           +   . N.D.
Dog       .    .    . +      +       +    . N.D. .     +    . N.D. - . N.D.
Cat     .   .    .     -      .     -    .    +    .   -    .   +    .   -   .N.D.
Human    .     .     . +  .   +  .   +    .   +    .   +    .

Monkey    .        .  . +  . +    .   -   . N.D. .     -    .   -    . N.D. . N.D.
HIorse    .    .              -         .  -  . -  .   -    .   -    . N.D. . N.D.
Cow.      .    .       -      -  .    -   .   --   .   +    .   -    . N.D. .     -
Sheep     .      .      +     +- +                      ?       - i

Goat.                      .  - - ?           +    .   +    .   -    . N.D .    N.D.
Chicken        .    .        +.      +    .   +    .   +    .   +    .   - . +
Turkey    .    .    . +    . +    . N.D. . N.D. . N.D. . N.D. . N.D. . N.D.
Goose     .    .    .      .  -   . N.D. . N.D. . N.D. . N.D. . N.D. . N.D.
Pigeon    .    .    .  -   .  -   . N.D. . N.D. . N.D. .        +    . N.D. .    +
Duck Anas mo8cant .                           + +                        .        -D.

Anas boschas  .      .  -

Frog Hyla arborea   .         +               +    .   +    .        . N.D. . N.D.

Bufoviridis   .  -   .  -   .

+ = positive.  - = negative. N.D. = No data. * = reported as + by Eddy et al. (1958a).

Winocour, 1959a), and if available in quantity would be the most suitable for
haemagglutination. Because of availability, guinea-pig red blood cells have been
used in all the following experiments.

The effect of pH

In order to test the effect of pH, haemagglutination was carried out in 0.15 M
phosphate buffer at different pH from 5.4-8.4. At the higher (7.7-8.4) and lower
pH (5-4 and 6.0), there was not a complete sedimentation of cells in the buffer
control. It is clear (Table II), that the virus can agglutinate over a wide range of
pH, although there is a lower titre at the lower pH. These tests and all the
following experiments on the properties of the haemagglutinins were made with a
virus stock of line IL-3, obtained by harvesting the fluid from cultures of tumour
cells (Sachs et al., 1959b).

Adsorption and elution from red blood cells

Adsorption and elution experiments were carried out to determine the pos-
session by the virus of receptor destroying enzyme, and the speed of the reactions.
A final concentration of 2 per cent red blood cells was used with the stock virus
fluid diluted 1: 8. Adsorption was carried out at 4? C. and elution at 37? C. For
the experiments shown in Table III, after adsorption of the virus, the cells were
sedimented by centrifugation in a clinical centrifuge at 1500 r.p.m. at 4? C., and
then washed twice by resuspending in the same amount of saline and centrifugation
at 4? C. Each elution was for 1 hour at 37? C, followed by centrifugation at 37? C.
to sediment the red blood cells. The cells were then resuspended in the same
amount of saline for the next elution. After 4 such elutions the cells were tested
for their ability to again adsorb virus.

Ectro-
melia

HAEMAGGLUTINATING SYSTEM OF POLYOMA VIRUS

I         I        I         I         I        I         I         I              -+

aq

-H-H I I I -Hf-H

00

co            +     +

-H-I    +I     +

o -F-I-+++++
esl- ++++

aq  ++++++

++++++
-F-F-+++++++

>+++ ++++

+++ ++++
++++++++

+o + ++ ++ +++
Ci + ++ ++ ++ +

++++ ++++
++++ ++++
1 ++ ++++++

00++++++++

++++++++
++++++++

Co++++ +++
*

+++++++

-F---F--
-FFFF----
CoFFF----

. . . . . . .

l I I I I I I I

0s+

00

+   +

o +1 1 1+1+1
-+   +

+   +

- +  I I I  ++ I
U1 +  +

+ +

+I I I ++++

C+++++

+   ++

Oo +  I +  I + +
*-+X

I + I +.++ +
I I I ++++
I I I ++++

++++

I I I ++++

* . . . . . .

(3O

.      II       I    I

--0

0

.       I    I   I    I

0

1-4
0

0

0

?
v

-H-HI I  I- - -H

m o

d o

F-

.,-I

"13

F-4

9

*-4

.0

.9

+
+

+
+
+
+

+
+
+
+
+F
+F
+F
+F
+F
-+
+F
+F
+F
+F
+F
+F
+F
+F
?F
+F

++

* ezb

ct

*t-I

Irp

H

E4

0
0
0
0
Ht
9
3

COD

1.

Eq
E--

IoII

O I I I I

0

+- +
0S  +

-+ +

o + I+I

0

-+ +

+ +

-+I ++  I

10

0+ +

00 ++ +

+ +

+ +

? +-

+++

ao +++ I

?? +++F I

+++

+++

I I
I   I
I I

+
I +

I-+

+

I +

+

I +

+

I +

+

+
I +

+
+

I +

+
++
+ +

++
++
+ +
+ +
++
++
++
++

++
++
??
++

ax
oG

1I

C;

f0
0

0
0

0
0
*- t
0

ll

:
c;

0
C3
0
0

Ct

>Q.

)*~ -e

~ 0

*0!;

EH

+

+

+

+

ca +

+
-+

- +
-+

~+

++++++++
-F-F++++++++F-
++++++++--F-

0
o

*  .  .  .  .  .  . * ;,

o~
?~ ~ ~

? " ? ?  4 ?

la
10.
0,

*     o
.   C ;

.d

, .0

. -

0

O bo

p ._

Q<

C)

269

ea
(M

oo

* . ? o

.

1.14 + + + + + + + +

M. FOGEL AND L. SACHS

The results given in Table III show that saline washing does not remove the
haemagglutinins from the red blood cells, and that even after 4 elutions at 37? C.
there are still some agglutinins attached to the cells. They further show that the
cells used for elution can again adsorb virus, so that polyoma does not seem to
have a receptor destroying enzyme such as is found in influenza, mumps and
NDV (Hirst, 1952).

To determine the speed of the reactions, the suspension was sampled at various
intervals after the beginning of adsorption and elution. Each test was made in a
different tube, the suspension centrifuged at the appropriate times, and the

U204

I

ADSORPTION
-0 0

oN                  I

0      0

\              ~~~~~~~~~~~~~~~~~~~~~~~~~~~~~~~~I

of             I
I  ?XsI

I I0

K0

,..,,  , . ,  , , ,, ~~~~~~~~~~~~~~~~~~~~~~~~~~~~~~1

1'2'   5'   10' 15' 20' 30;iin 1     2  3    16hr.

0       0   0

ELUTION
0 00

r

1'2'  5'   10' 15' 20' 30m;n. I 2 3hr

FIG. 1.-Adsorption and elution curves for polyoma haemagglutinins on guinea-pig red blood cells.

haemagglutination titre assayed. The results (Fig. 1) show a rapid adsorption
and a rapid elution. The titre dropped from the original 2048 to 64 after an
adsorption time of 10 minutes, and during elution, the titre rose to 512 after
1 minute and to 1024 after 10 minutes. A rapid adsorption was also found by
Hirst (1942) for influenza virus, although elution with influenza was not as rapid
as with polyoma. The polyoma did not show spontaneous elution at 4? C.
Sedimentation at 105,000 g.

It was found that supernatant fluid after centrifugation for 1 hour at 105,000 g
produced tumours when inoculated into newborn Swiss mice. Experiments were
therefore carried out on the sedimentation of the virus at this speed. Stock virus
was first centrifuged at 9000 g for 10 minutes in a Servall Angle Centrifuge, and
the supernatant then centrifuged in a Spinco Model L Preparative Ultracentrifuge

1024

512

256

128

64

32

w

-

-

z
0

z

I-

3

.J

CD
CD
4-
w
4

16

I

a

.     .   .   .   .   .                                                                  -        -

L

I  I  I I  Da

270

HAEMAGGLUTINATING SYSTEM OF POLYOMA VIRUS

with a No. 40 Rotor at 40,000 r.p.m. average - 105,000 g. The supernatant and
sediment were sampled for haemagglutination titre from 12 c.c. of virus stock after
1 hour, and from another 12 c.c. after 3 hours centrifugation in the Spinco. The
sediment was resuspended in 5 c.c. of medium without serum.

The results, Table IV, show that after 1 hour haemagglutinins have sedimented.
Samples of 1 c.c. from supernatant after 1 hour, and from the top 2 c.c., the
bottom 10 c.c. and the resuspended sediment after 3 hours centrifugation, were
inoculated intraperitoneally into adult Swiss mice. Haemagglutination inhibition
antibodies in the pooled serum from 2 mice per group were determined 14 days
after inoculation and these gave titres of 1: 1280, 1280, 1280 and 2560 respec-
tively. The supernatants thus still contain some virus particles. The size of the
particles and possible existence of aggregates is being checked by electron micro-
scope observations. Filtration of stock virus through a Selas 02 candle filter did
not decrease the haemagglutinin titre. The production of tumours by the super-
natant after 35 minutes at 105,000 g has also been reported by Buffett et al.
(1958).

Ultraviolet inactivation

The inactivation of polyoma by ultraviolet was examined in order to determine
the degree of inactivation of the haemagglutinating particles and to compare this
with effects on infectivity. Infectivity was measured on tissue cultures and in
animals. Stock virus was centrifuged in a Servall Angle Centrifuge at 9000 g for
10 minutes, and for each test, 1 c.c. of the supernatant was irradiated in a watch
glass with a 15 watt Philips TUV germicidal lamp placed at a distance of 24 cm.
from the virus containing fluid. This gives a radiation intensity of 30 ergs/mm.2/
second. The watch glasses were agitated every 10 minutes, and before titration
any medium loss by evaporation was added to make the original 1 c.c. The
haemagglutination titres obtained after various exposures (Table V) show that
there was a decrease of one dilution after 60 minutes, a further decrease by one
dilution after 100 minutes, and that there was still some agglutination after
150 minutes.

To compare the effect on haemagglutination with effects on infectivity, virus
after various periods of irradiation was inoculated onto mouse embryo monolayers.
The monolayers were grown in petri dishes in a medium of 0.5 per cent lactalbu-
min hydrolysate in Earle's saline and 20 per cent horse serum, in an incubator
with a humidified atmosphere containing 10 per cent CO2. Each test was made on
a pool from two petri dishes, and each petri dish containing 4 c.c. of medium.
0 3 c.c. of virus was adsorbed for 3 hours, without washing, and the medium changed
at 2, 5, 8 and 11 days later. The haemagglutination titres per 0-5 c.c. were deter-
mined on days, 5, 8 and 11, and the cultures were also examined for a clear cyto-
pathogenic effect. It can be seen (Table VI) that virus irradiated for 30 minutes
before inoculation on to monolayers gave after 11 days growth a haemagglutination
titre of 256 (in contrast to a titre of 2048 for non-irradiated virus) and that after
60 minutes irradiation the titre was only 32 in comparison to 16 for normal tissue
culture fluid. After an exposure of 90 minutes or longer there were no haemag-
glutination titres higher than those of the normal tissue culture fluid. Since the
yield of haemagglutinins in tissue cultures is correlated with the amount of
infective virus in the inoculum, these results show that, as in the case of influenza

271

M. FOGEL AND L. SACHS

(Henle and Henle, 1947), infectivity of the polyoma virus is more sensitive to
ultraviolet than haemagglutination. There was also a correlation between haemag-
glutinin yield and the cytopathogenic effect on the culture (Table VI).

The infectivity of the irradiated virus was further measured by determining
the haemagglutination inhibition antibody titre at 2 and at 4 weeks after inocu-
lation of 0.05 c.c. subcutaneously into 2-day-old Swiss mice. Each test was made
with the pooled serum from 2 or 3 mice, and the results are shown in Table VI.
The increase in titre between 2 and 4 weeks indicates that there may still have
been some infective virus after 150 minutes' irradiation.

Temperature inactivation

In order to determine stability at different temperatures, experiments were
carried out on the temperature inactivation of the haemagglutinins and infectivity.
The tests for infectivity and cytopathogenic effect were made in the same way as
for the ultraviolet experiments, with samples from the same virus stock. There
was no decrease in haemagglutination titre (Table VII) after 30 minutes at 56? C.,
a decrease at higher temperatures, and no significant titre with the method used
after 30 minutes at 66? C. or 15 minutes at 70? C. The infectivity tests on the
cultures (Table VIII) showed no decrease in yield of haemagglutinins after 30
minutes at 56? C., a decrease at higher temperatures, and that there was still
some infectious virus after 30 minutes at 66? C. and 15 minutes at 70? C. The
existence of some haemagglutination titres lower than the control of 32 in column 2
of Table VIII can be explained by the inactivation at these temperatures of the
agglutinating antibody in the horse serum of the medium.

The haemagglutination inhibition titres after inoculation into mice (Table VIII)
also show the high temperature stability of the virus under the present conditions,
and that there may still have been some infective virus even after 30 minutes at
70? C. The existence of some infective virus after 30 minutes at 70? C. has also
been reported by Eddy, Stewart and Grubbs (1958b). In tests on the stability of
the haemagglutinin at 37? C., it was found that there was no loss of titre after 8
weeks at this temperature.

It can be seen from the above that whereas in the ultraviolet inactivation tests
infectivity was clearly more sensitive than haemagglutination and this has also
been found with inactivation by formalin (Sachs and Fogel, unpublished), such a
clear distinction between these two properties was not found in the temperature
tests.

The haemagglutinins from different tumours

Virus has been isolated from different polyoma induced tumours, and the anti-
genic similarity of the haemagglutinins determined by cross inhibition tests. The
lines used were IL 1-6, and these consist of isolations from mouse parotid tumours,
mouse mammary adenomas, a mouse mammary adenocarcinoma, and a rat kidney
sarcoma. It was found that these virus isolates all gave cross haemagglutination
inhibition, so that the haemagglutinins from different tumours have common
antigens. Cross inhibition was also obtained with virus isolated from a C3HGr
leukaemic mouse (Sachs et al., 1959b). These results provide further evidence
that more than one type of tumour may be produced by the same virus.

272

HAEMAGGLUTINATING SYSTEM OF POLYOMA VIRUS

c0

0-

_n 0

I I I I I I I I I I I I

I I I I I I I I I I I I

I I I I I I I I I I I I

10

o I
0

I   I + I   I   I   I   I   I   I   I

00

I +++   II  I

++++ +++

++++ ++++++++
++++ +++

++++?+++++ I
+++++++++

+++++++++ ++

+++++++++

+++++++++ +

+++++++++++
+++ ++++++++

++++++++++++
Z ++++++++++++

++++++++++++

- ++++++++++++

+++++++++ +
+++++++++++

q ++++++++++++

+++++++++++

+++++++++++

X+++++++++ +++

+++++++++++

++++++++++ +
X+++++++++~+++
+++++++++ +

+++++++++

c++++++++++[+
+++++++++

o;.~

0..

ez

0
V

I.

DH

o  o

S5 S ,;o-oooooooo

0

z.,  _o

._          4   4"q-

L-4- 4

.--,B

~0      " -1

-4       0

,g -

-4 0s d

50 *      --?

4a C.) t a O,

bC - >4 0

03   -
t.5 (D1

E-o

0 _ r

0

~o

0

0

- Q 4-

00

0

0 <

2 *D

S oP)

00000000C
00000000

z

+  E + ll  ll

z

1 I  I o   I  I  I  I

Hs  oi  o  - - -   -

cq +4  e

E1  _- c=

10   . . .

~~   ~ ~ ~       *

5  b  .   0  c   0   00  c   0 1

0d      C 0   ' 0 0

O ; @em   aq all

* 0

.

4--l t g ?          _e

0              0
zz

273

-4
.._

Qo

e0

n,

0

He

()

1.

0
C5
d

1.

C5

0
O

4D
0
,X,
*
*

10

-4
4D

* .

bO
o,.i

.I0
+4

+

?o+

I4

Q

I d=

0_

I

e

0        .

M. FOGEL AND L. SACHS

0
0

e0
0

? . . . . . . . . . . .
I I I I I i I I I I I I

co ++

-- +-+     I  I  I  I  I  I  I  I  I  I  I

0 ++
X ++

g +++ I I I I I I I I I I

++

> ++++ I I I + I I I I I

++

C ++++
I'M ++++

++++
10 q-

I + I ++I I I

++++++ ++

++++    ++ I I I
+++++ + +
+++++++++

+++++++++ I I I I
+++++++++
+++++++++

+++++++++III
+++++++++
+++++++++

+++++++++ I I +I
++++++++++
+++++++++

X++++++++++II

+++++++++

++++++++++

+ ++++++++ I I +
+++++++++

+++++++++  +

cS+++++++++1 !l+ l

+++++++++  +

.     .   .   .  .   .   .   .   .   .   .
0

10 to co   o  o Co  o 0 0

Co CO  O CO  O Co Co  o t- Eo-

* t

Q.o

*E00
EH

0      P.-
o     ",
C)
Q

(d )    _

C)   0

o          I

40      0)

-0

1 *

d 0

o

r- d

00

* V-

E

ibS o?

W 0

-4

-4'-  Go

Go ED d
O -8: 4

ooooooooos-

ll l,,10 0 l1o ?q 10 -4 e.

c0 c1 oo cc -o cl 10 10c in E

No oq o o oN oN ow co WCor

M          CO CO COr X

* . . . . . . . . .

++++,-            I  I  I  I  I

M ++ I I I I I I I I
1   ++     I I     I I    I I I

C- O O O10 1 -
- 00001001

1000104  Mm

o-i -q

1~4 -
0

C))

4.0.

0-  ~

O O 't uq " r ," ,,,,s
? Co

GL 01i -    CO

40     0   0 0

0D 2

14  0

H   _

to O1 4 I4 CZ co O

Zr1 co co  o  o  o o 1o

274

o,

.0

'40

.1

IR.

H.I

O

C5

O
C)
0

EI

0
oo

P11

0o

-4

H
*

*

40D

0

._z
z

ci

p.4

0t

+40

,;
0d

Crj;

40m

400H

40

I 40

OQ
0._

-40
0

H

HAEMAGGLUTINATING SYSTEM OF POLYOMA VIRUS

Haemagglutination inhibitors in extracts of normal and tumour bearing animals

It has previously been found that extracts of tumours taken straight from the
animal did not give haemagglutination, whereas high haemagglutination titres
could be obtained from tumour cells in culture (Sachs et al., 1959b). The absence
of haemagglutination by extracts of tumours from the animal may be due either
to a low haemagglutinin content or the existence of inhibitors. In the present
study the latter possibility has been examined. Organs from normal mice, tumour
bearing mice, and the tumours themselves, were homogenized with a motor driven
homogenizer, centrifuged for 10 minutes at 9000 g in a Servall Angle Centrifuge,
and the supernatant tested for haemagglutination inhibition. The tests were
made in duplicate using supernatant unheated and heated for 30 minutes at
56? C., and the results compared with the effects of normal and immune serum.
Three series of tests gave similar results and the data from one of these are given
in Table IX.

The results show that inhibitors to a titre of 1: 80 exist in organs from normal
mice and that these are destroyed by heating at 56? C. for 30 minutes. Antibodies
in immune serum, and the inhibitors (up to 1: 40) in normal serum are not
destroyed by this heating. In addition to the heat labile inhibitors, the extracts
of organs from tumour bearing mice and from the tumours themselves also have
high titres of antibodies, and both these, particularly the latter, could explain
why extracts from tumours do not give haemagglutination. The presence of these
inhibitors is also of significance in the actual isolation of viruses from tumours.

Haemagglutination inhibition antibodies and turnour formation in mice and hamsters

It has previously been shown that newborn mice inoculated with virus develop
haemagglutination inhibition antibodies, and that there are high inhibition titres
when palpable tumours are formed (Sachs et al., 1959b). Further experiments
were therefore carried out on haemagglutination inhibition antibodies and tumour
formation in mice and hamsters. The newborn animals used were less than 24
hours old. Mice were inoculated subcutaneously with 0-05 c.c. and hamsters
subcutaneously with 0.1 c.c. of virus suspension. Curves for inhibition antibody
in three separate mice, tested at various intervals after virus inoculation into
newborn, are shown in Fig. 2. In two of these with large tumours, the antibody
titre increased up to the time of appearance of palpable tumours, and in the third,
which had an unusual small slow growing tumour in the foot, the titre has con-
tinued to increase after the appearance of the tumour until it has so far reached
1: 20,480. In the mice with the large tumours the same antibody titre was reached
where the tumour appeared at 10 weeks and where it appeared at 23 weeks.
Haemagglutination inhibition antibodies were therefore present in the mice
during the entire period of growth after virus infection. Further data on this
point are given in Table X, and these also show that the presence of palpable
tumours is always associated with a high titre of inhibition antibodies.

In hamsters, although inhibition antibodies are also present after virus in-
oculation, the titres are lower in animals with palpable tumours than in the case
in mice (Table X). Palpable tumours in hamsters develop earlier than in mice
(Eddy et al., 1958c). The earliest palpable polyoma induced tumours in our
hamsters developed about 4 weeks after inoculation in contrast to about 9 weeks
before the earliest palpable tumours are found in mice (Sachs et al., 1959b).

275

M. FOGEL AND L. SACHS

C

R O' + +  + +

+ +   ++

+2 0++  + +

+++
o ++   ++

++   ++

+++

o      ++

++   ++
o+ ++

+ +
+1   ++
O ++ ++
++ ++

O + +++

+ + + +

4 ++  +++
O+ +

+  ++
++   ++

+  ++
++       +
+   ++

+ +  + +
++   ++
o++

++

+ +  + +
+ + + +

++

++
_ + +  ++
O      ++  +

++   +

++
0      ++  +

++    ++
++   ++

+
+

e I +    +

+   ++

+
+

O I +    +

+   ++

+ +   +
+     +
- 1+

+

i +

C,SZ C) S

_~

o.

0 .~
ez

++  ++  ++  +
++  ++  ++  +
++  ++  ++  +
++  ++  ++  +
++  ++  ++  +
+ +  ++  ++  +

++   +  +

II  ++  i+  +

++   +  +
++      +

I I  ++  I?  +

++   +  +

+      +

I I  +  II  +

+      +

+

I I  +      +

+      +

+
+

I  +  I  I  I  I  +

+
+

I +  I  I I  I  +

+          +

+

+          +
+          +

+

+          +
+   +      +
+          +
+          +
1+  1+  I  +
+ +        +

+     +
+      +
+     +
+     +

I+    I+

+     +
+     +

I+    I+

+     +

0

0

o

4a,

?
+
+

+
+
+
+
+
+
+
+
+
+
+
+
+
+
+
+
+
+
+
+
+
+
+
+
+
+
+
+
+

+
+
+

?

+
+
+

+
+

I I
I I
I I
I I
I I
IIl

I            I
I            I

1+    ++    II

1+    II    II

+

+

1+    II    II

+

? ? ?

? ?ofl ? ?

"? ?o ? ?o "?

? ?kL?C0 ?

10

?

I._

Cs 0

M

0
C)

0
0

?
Co.
E
?

F-
C

a4
It

C
S

4a
fr

o~.
o~

q4~
*s
*0o
0O
0

0 s

H

- .m

o                00

CE ?

;3                1

?'- (D

276

-1
-1

5

4
D
1
4
-4

I

-4
4
-1

I,
4

D
ID
-1

9
9

:0
4

1

HAEMAGGLUTINATING SYSTEM OF POLYOMA VIRUS

TABLE X.-Haemagglutination Inhibition Titres at Various Intervals After Virus

Inoculation into Mice and Hamsters

Mice

A-                     .

Age at
time of

Palpable inoculation
tumours   (days)

_-~2 2

-       N.B.
-       N.B.
-       N.B.
-       N.B.
-       N.B.
-       N.B.
-       N.B.
-       N.B.
+        N.B.
+        N.B.

_-~2 2

+         2

+        N.B.
+        N.B.
+        N.B.
-       N.B.
N.T.      N.T.
+        N.B.
+        N.B.
-       N.B.

Hamsters

Age at
time of

Palpable   inoculation
Titre 1:    tumours       (days)

2560          -           7
2560          -            8
2560          +            2
5120          +            2
2560          +           2
2560          +            2
2560          +            2
2560          +           2
1280          +           5
2560          +           8
2560          +           3

N.T.        N.T.         N.T.
1280          +           3
640          +           5
640          +           5
640          +           3
2560          +           3

640          +           3

N.T.        N.T.         N.T.
N.T.        N.T.         N.T.
N.T.        N.T.         N.T.

* Litter of 4 mice pooled. ** Litter of 7 mice pooled. N.B. = Newborn. N.T. = Not tested.
+ = Present. - = Absent. Each test represents the result from a different animal.

TABLE XI.-Haemagglutination Inhibition Antibodies in AKR Mice

Age in
Number of       weeks at

mice          1st test

5
3
1
1
1
1
1
1
1
1
1
1
1
1
1
1
1
1
1
N.T. = Not tested.

13
15
15
17
17
23
23
32
32
32
32
32
32
34
34
35
35
36
36

Antibody titre 1:

-A             -

2nd test    3rd test
4 weeks     8 weeks
1st test     later      later

20          20          20
20          20          20
10          20         20
20          40         40
20          20          20
20          40          20
20          40          20

320        5120         N.T.
640        5120        2560
1280        2560       2560
1280        5120       2560
1280        5120       2560
1280        5120       2560

20          20          20

1280         N.T.       N.T.

20          10          20

20         N.T.        N.T.
10         N.T.       N.T.
2560         N.T.        N.T.

Time after
inoculation

(weeks)

2
3
5
5
5
7
7
9
11
11
11
12
12
13
13
14
15
16
20
22
22

Titre 1:

640*

2560**
2560
5120
5120
5120
10240
10240
20480
40960
10240
10240
40960
? > 20480

40960
20480
20480
N.T.
. >5120

40960

5120

277

M. FOGEL AND L. SACHS

Hamsters also have a different distribution of tumour types (Eddy et al., 1958c),
an observation that we have confirmed. The difference in inhibition antibodies
between mice and hamsters does not seem to be due to the existence of an initially
weak immune response in the hamster, since animals inoculated at 7 or 8 days of
age (Table X) have antibody titres of 1: 2560 after 2 or 3 weeks. The difference
may be due to differences in cell virus relationships and the production of haemag-
glutinating particles. In addition hamsters may not produce titres higher than
1: 5120, and there may be more antibody adsorption in vivo in hamsters than in
mice. These possibilities are now being examined.

2c

IC
W

$-

0

1-

p
0
m

z
4

2 4   6 8 10 12 14 16 18 20 22 24 26 28 30 32 34

TIME IN WEEKS AFTER VIRUS INOCULATION.

FIG. 2.-Haemagglutination inhibition antibody titres in relation to the appearance of palpable

tumours in three mice inoculated as newborn with polyoma virus.

Haemagglutination inhibition antibodies in AKR mice

The existence of haemagglutination inhibition antibodies against polyoma
virus in some AKR mice 32 weeks of age or older has already been reported (Sachs
et al., 1959b). These animals have now been further studied in order to obtain
additional information on the existence of these antibodies in AKR, a strain of
mice with a high incidence of spontaneous leukaemia. The results of two sets of
tests, 4 and 8 weeks after the original titrations, are given in Table XI. These
show that the animals with antibodies were also positive at the later tests.

All the mice have been kept as breeding pairs, and the 6 mice, 32 weeks old
when first tested and found to have antibodies, are all litter mates belonging to
the same family. In addition however, the 5 negative mice 13 weeks old at the
first test, are litter mates derived from one of the pairs positive at 32 weeks. If
the parents had been positive from the beginning, it may be assumed that their
offspring should also be positive (Sachs et al., 1959b, and unpublished), so that
it appears that the mice positive at 32 weeks were negative at an earlier age. Four
week old litters (7 animals tested) born from the negative mice 13 weeks old at the
first test were also negative. Furthermore, both of the other positive mice shown
in Table XI (one 34 and one 36 weeks old at the first test) have negative litter
mates. These litter mates have been kept since birth in the same animal cage.

278

I

HAEMAGGLUTINATING SYSTEM OF POLYOMA VIRUS

If the presence of inhibition antibodies in AKR were due merely to polyoma con-
tamination in our laboratory, it would be expected that both members of the
litter should have inhibition antibodies, since the presence of animals in the same
cage seems to be the best means of ensuring contamination (Sachs et al., 1959b,
and unpublished). All AKR mice have been kept by us since their introduction
in a different room from animals inoculated with polyoma virus. The existence
of inhibition antibodies against polyoma in some AKR mice has also been
observed by Rowe et al. (1958), who seem to interpret their results as due to
contamination of the animals with polyoma.

DISCUSSION

The present studies have further shown the value of combining the in vivo
with an in vitro approach in defining the properties of a mammalian tumour virus.
The data on the haemagglutinating spectrum, and the rates of adsorption and
elution, may serve as markers for the finding of mutants of the polyoma. Regarding
the epidemiology of this virus, its high temperature stability would be a potent
factor in determining its ability for survival and re-infection. In common with
non-tumour forming viruses, infectivity is more sensitive than haemagglutination
to ultraviolet inactivation, and although polyoma has a high resistance to ultra-
violet inactivation, this could be a suitable method for virus inactivation and the
maintenance of antigenicity. However for this latter purpose formalin has been
found very satisfactory (Sachs and Fogel, unpublished).

The finding of haemagglutination inhibition antibodies in both mice and
hamsters throughout their entire period of growth after virus infection, and the
demonstration that tumour cells can continue to produce haemagglutinins (Sachs
et al., 1959b), makes it possible to determine the presence of this virus in tumours
by testing the serum of animals for inhibition antibodies. An equivalent method
has also been of value in studies on tumours induced in rabbits with papilloma
virus (Rous, 1935).

It appears from the results obtained with AKR mice that some animals of
this high leukaemic strain may develop inhibition antibodies against polyoma.
Although it has been experimentally established that animal contamination with
polyoma can take place (Sachs et al., 1959b), the data presented, which include
the observation that antibodies are found in older but not in young mice, make
it unlikely that all the results with AKR are merely due to laboratory con-
tamination. The possibility exists that virus activation may occur in AKR mice.
Activation of a provirus may thus actually be demonstrable with a tumour virus,
and further experiments in this direction are under way. In contrast to activa-
tion towards lytic action, i.e. a state that causes only cell destruction, as is found
in lysogenic strains of bacteria, this would be activation to a stable cell virus
relationship that under the appropriate conditions causes cell proliferation, i.e.
the development of tumours.

SUMMARY

A study has been made of the haemagglutinating system of the polyoma virus
with particular reference to the properties of the haemagglutinating particles, the
formation of haemagglutination inhibition antibodies in relation to tumour
formation, and the existence of such antibodies in AKR mice.

279

280                     M. FOGEL AND L. SACHS

The polyoma can haemagglutinate in the cold (4? C.), the virus has a specific
haemagglutination spectrum when tested with red blood cells from various species,
and haemagglutination can take place at pH from 5.4-8.4.

Experiments on adsorption and elution have shown that the virus does not
seem to possess a receptor destroying enzyme, that there is a rapid adsorption
at 4? C., and a rapid elution at 37? C.

Sedimentation studies at 105,000 g have shown that haemagglutinins can
sediment after 1 hour of centrifugation, but that even after 3 hours the super-
natant still has some virus particles.

Ultraviolet irradiation experiments have demonstrated that in common with
non-tumour forming viruses, infectivity is more sensitive than haemagglutination
to inactivation with ultraviolet.

Temperature inactivation studies have shown a high stability of both infec-
tivity and haemagglutination after 30 minutes at 56? C., and that there may
still be some infective virus after 30 minutes at 70? C.

Virus isolation from polyoma induced parotid tumours, mammary adenomas
and an adenocarcinoma in mice, and a kidney sarcoma in rats, gave cross haem-
agglutination inhibition, so that haemagglutinins from these different tumours
have common antigens.

It was found that extracts of normal mouse organs possess haemagglutination
inhibitors to a titre of 1: 80, and that these inhibitors are destroyed after 30 minutes
at 56? C. Extracts from organs of tumour bearing mice and from the tumours
themselves contain inhibition antibodies in addition to these heat labile inhibitors.

Haemagglutination inhibition antibodies were shown to be present in animals
throughout the entire period of growth after virus infection. In mice the presence
of palpable tumours was always associated with a very high titre of inhibition
antibodies, whereas hamsters with palpable tumours do not have such high titres.

Evidence has been presented to show that AKR mice may naturally develop
haemagglutination inhibition antibodies against polyoma virus.

We are indebted to the Winfield Baird Foundation for a grant in support of
this work.

BIBLIOGRAPHY

BUFFETT, R. F., COMMERFORD, S. L., FURTH, J., AND HUNTER, M. J.-(1958) Proc. Soc.

exp. Biol., N.Y., 99, 401.

BURNET, F. M. AND STONE, J. D.-(1946) Aust. J. exp. Biol. med. Sci., 24, 1.

EDDY, B. E., ROWE, W. P., HARTLEY, J. W., STEWART, S. E. AND HUEBNER, R. J.-

(1958a) Virology, 6, 290.

Idem, STEWART, S. E. AND GRUBBS, G. E.-(1958b) Proc. Soc. exp. Biol. N.Y., 99, 289.
Idem, STEWART, S. E., YOUNG, R. AND MIDER, G. B.-(1958c) J. nat. Cancer Inst., 20,

747.

ELLERMAN, V. AND BANG. O.-(1908) Zbl. Bakt., 46, 595.

GROSS, L.-(1957) Proc. Soc. exp. Biol. N.Y., 94, 767.-(1958) Cancer Res., 18, 371.
HENLE, W. AND HENLE, G.-(1947) J. exp. Med., 85, 347.

HmIRST, G. K.-(1942) Ibid., 76, 195; (1952) In 'Viral and Rickettsial Infections of

Man,' 2nd Ed. (T. M. Rivers, editor). Philadelphia (J. B. Lippincott Co.), Chapter 4.
OBERLING, C. AND GUERIN, M.-(1954) Advanc. Cancer Res., 2, 353.

Rous, P.-(1911) J. exp. Med., 13, 397.-(1935) Harvey Lect., 31, 74.

HAEMAGGLUTINATING SYSTEM OF POLYOMA VIRUS                 281

ROWE, W. P., HARTLEY, J. W., BRODSKY, I., HUEBNER, R. J. AND LAW, L. W.-(1958)

Nature, Lond., 182, 1617.

SACHS, L., FOGEL, M. AND WINOCOuIR, E.-(1959a) Ibid., 183, 663.

Iidem, HELLER, E., MEDINA, D. AND KRnLM, M.-(1959b) Brit. J. Cancer, 13, 251.
SALK, J. E.-(1944) J. Immunol., 49, 87.

SMADEL, J. E.-(1948) ln 'Viral and Rickettsial Infections of Man' (T. M. Rivers,

editor). Philadelphia (J. B. Lippincott Co.), Chapter 3.

STEWARD, S. E., EDDY, B. E. AND BORGESE, N.-(1958) J. nat. Cancer Inst., 20, 1223.
WINOCOUR, E., AND SACHS, L.-(1959) Virology, 8 (in press).

20

				


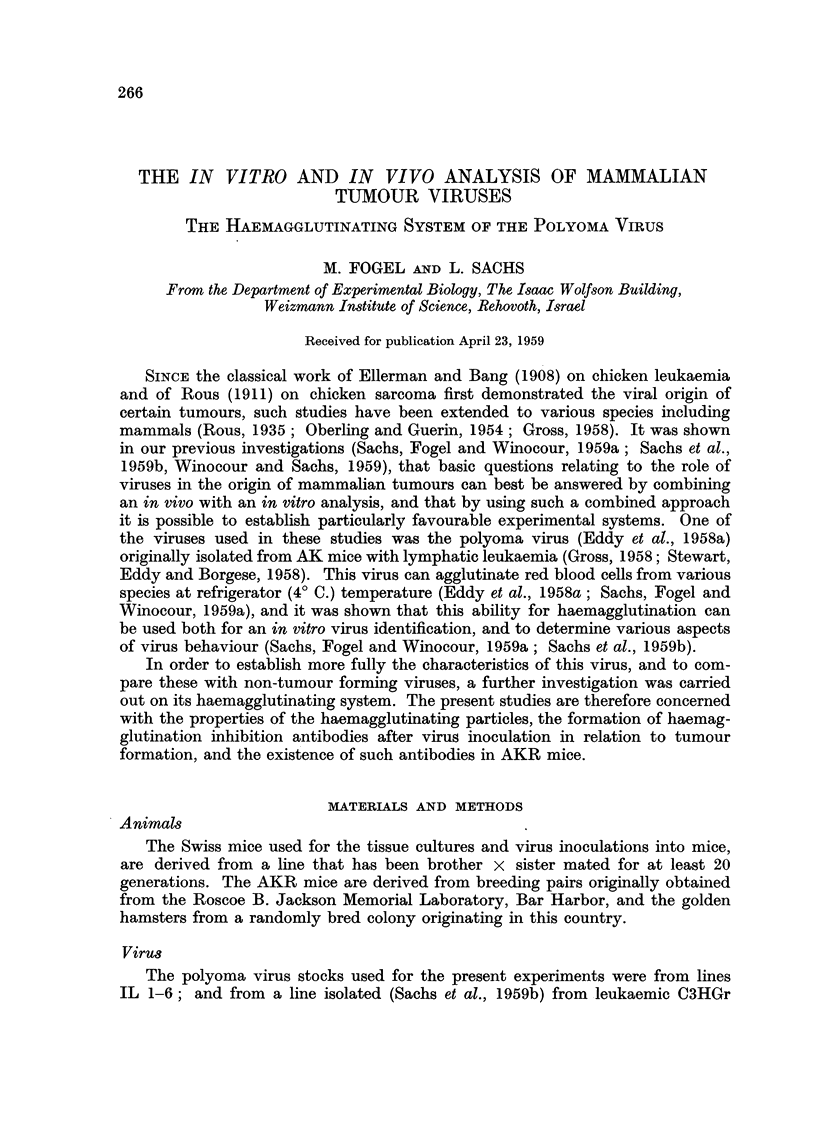

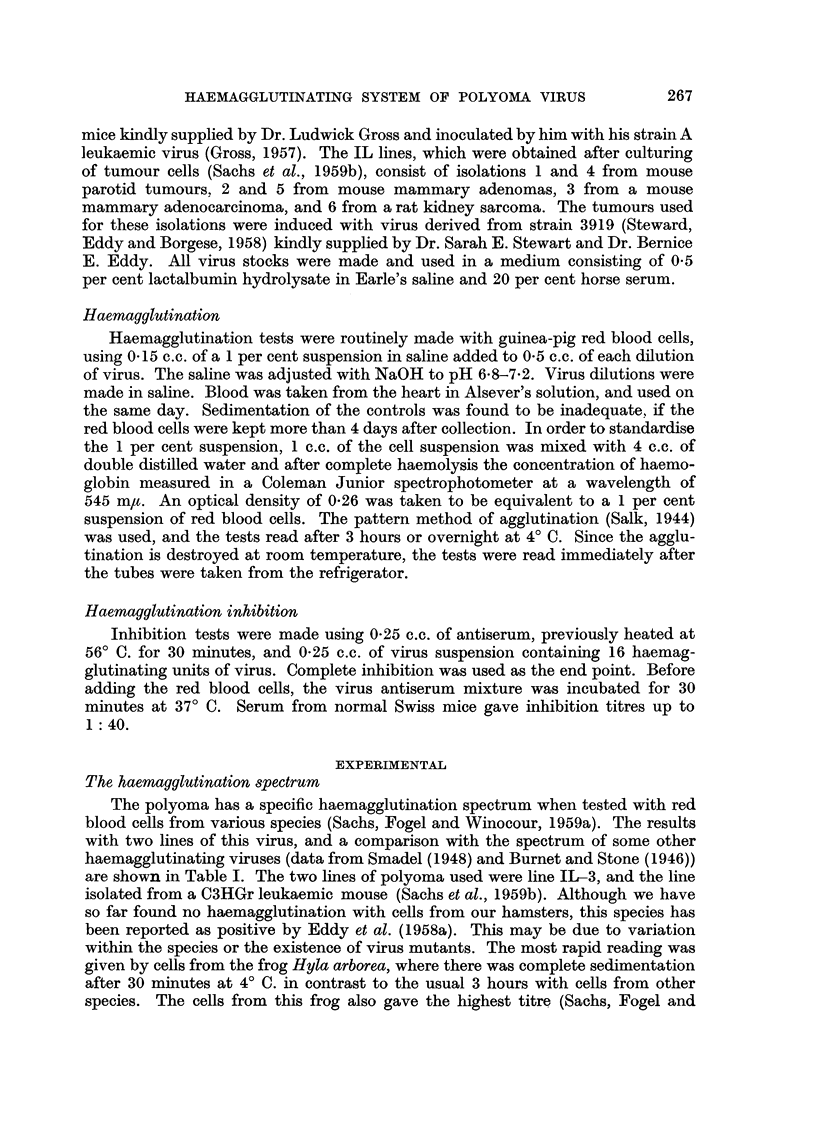

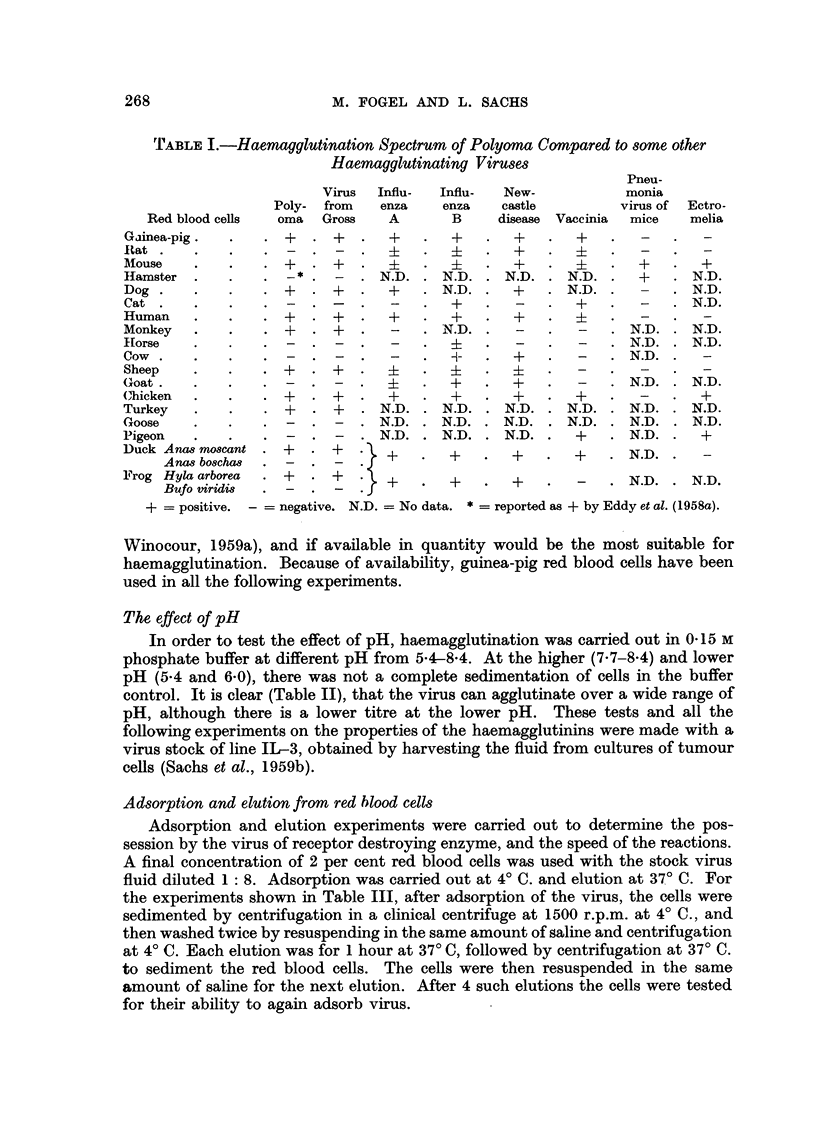

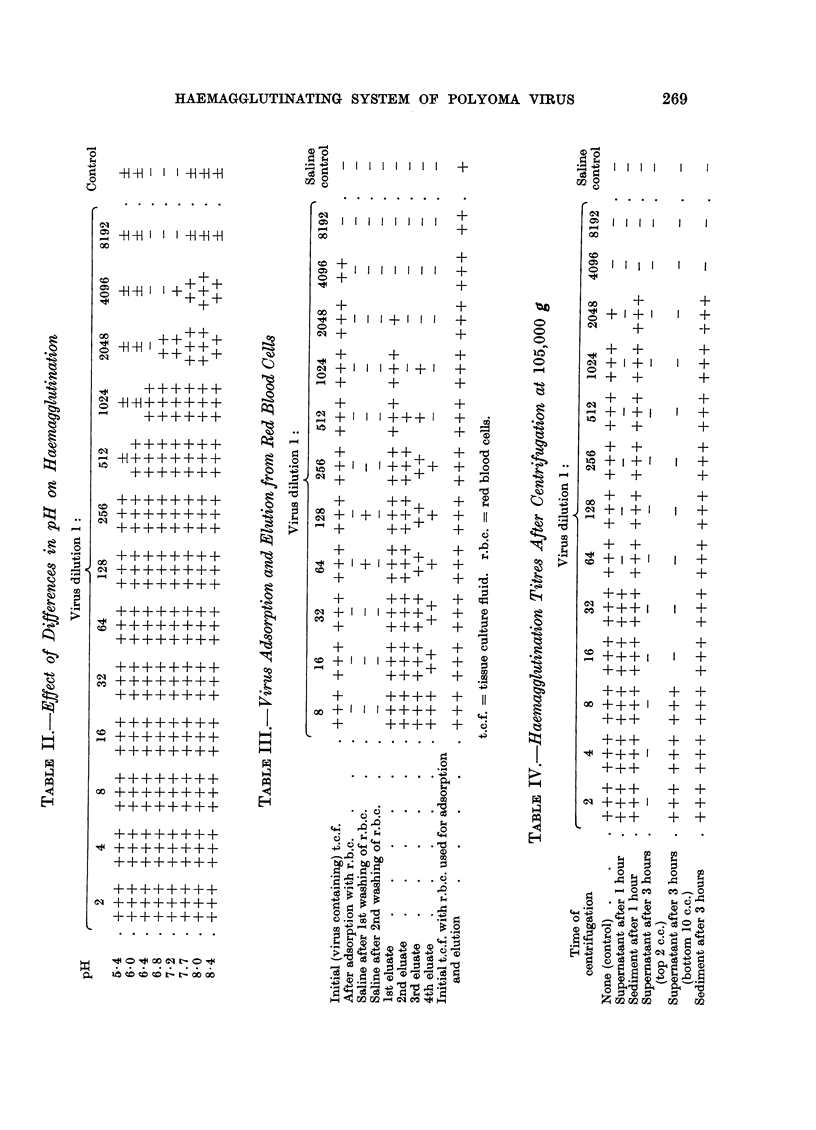

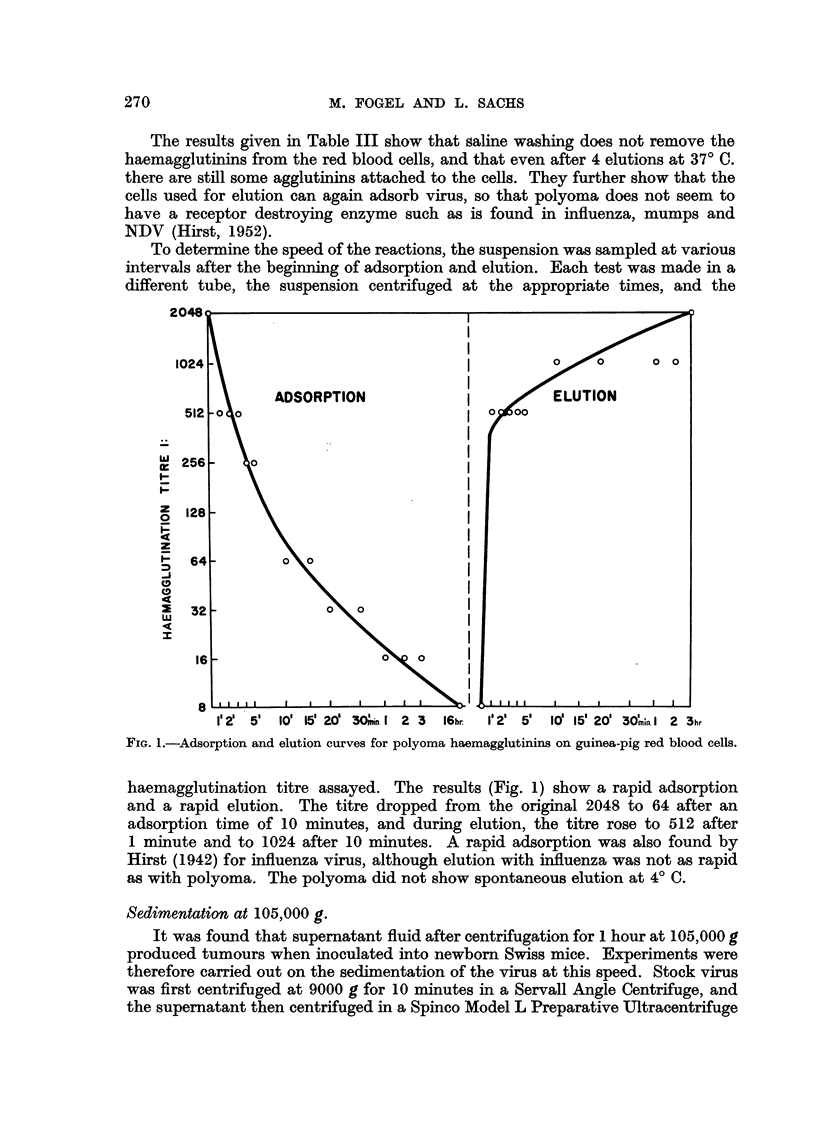

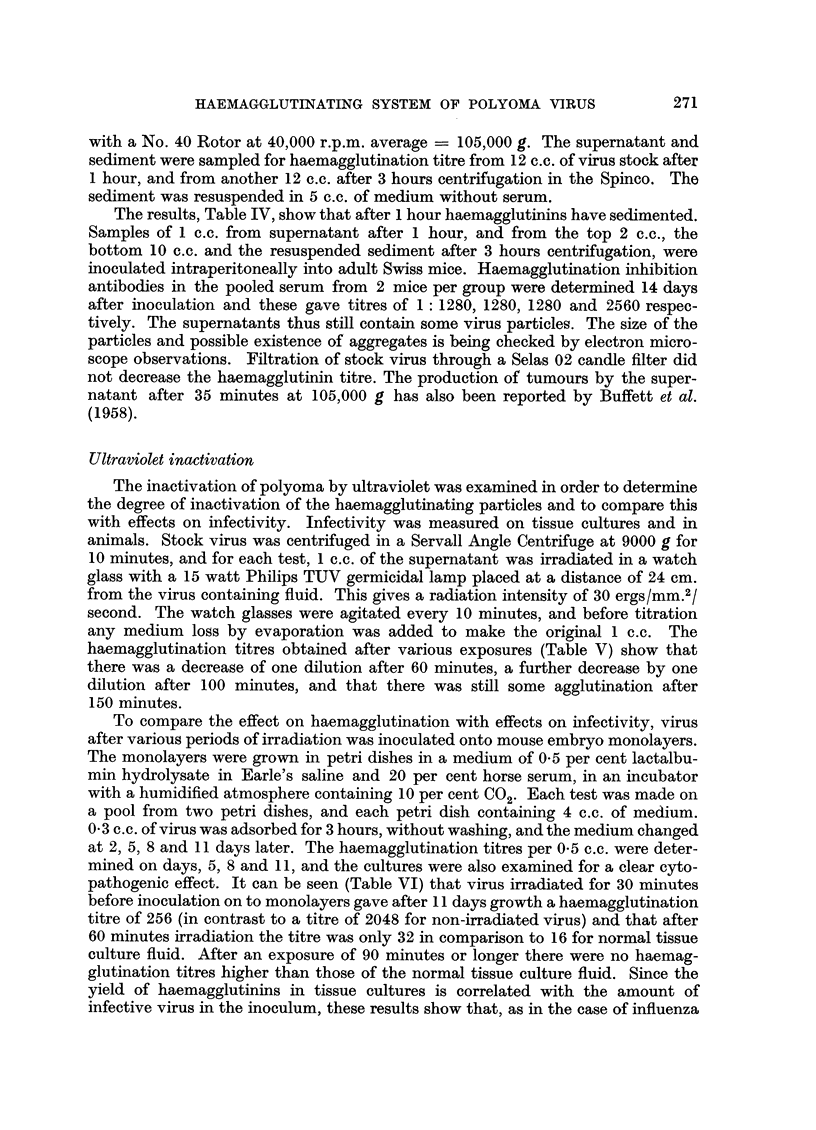

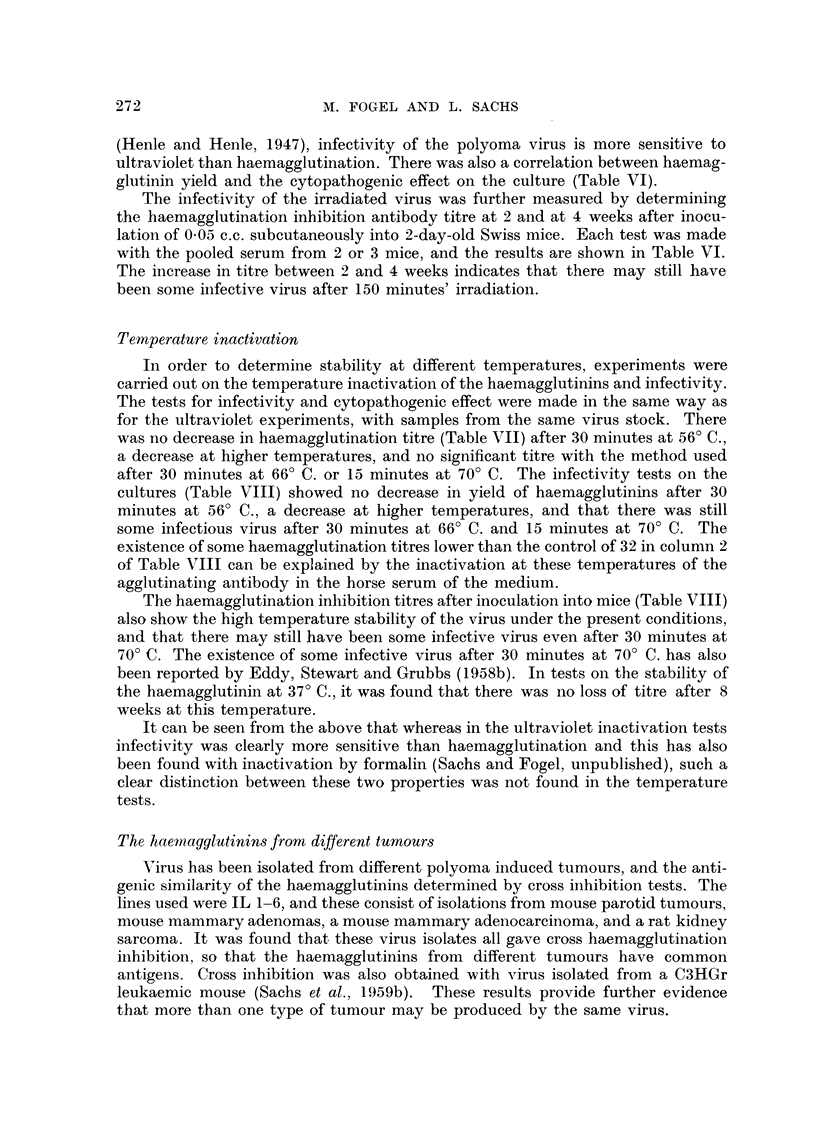

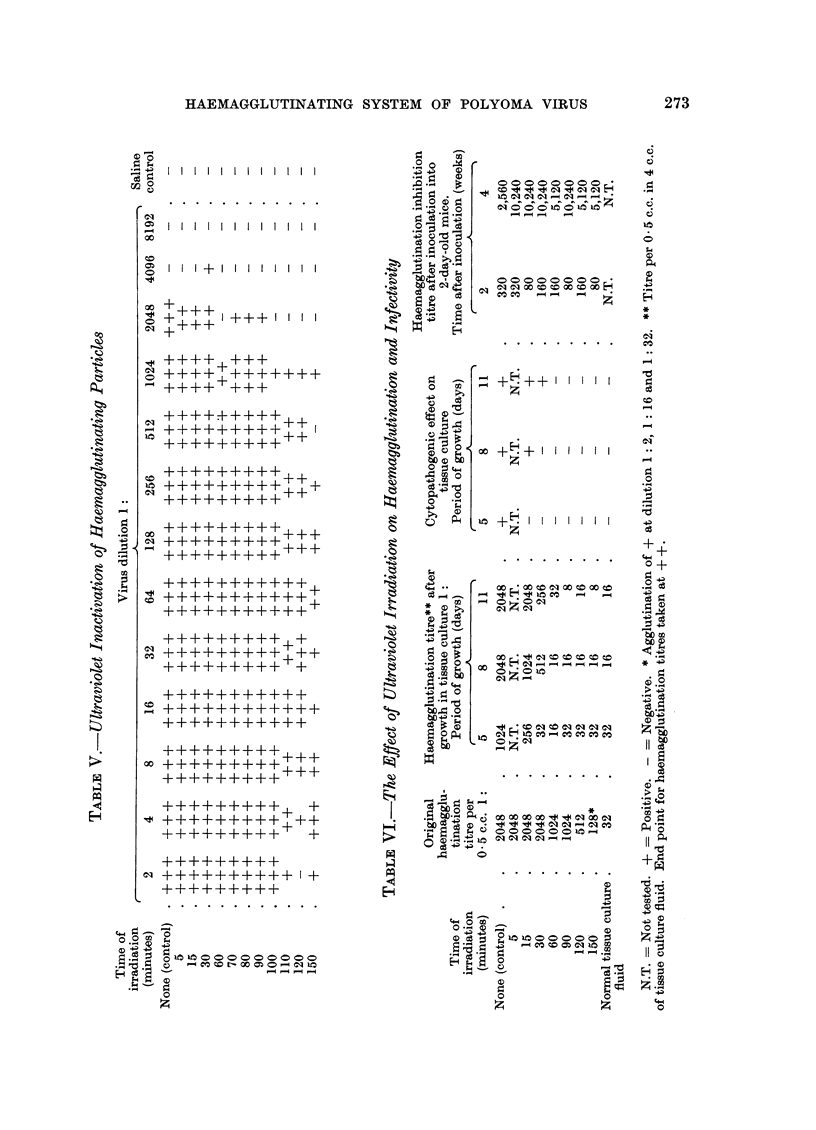

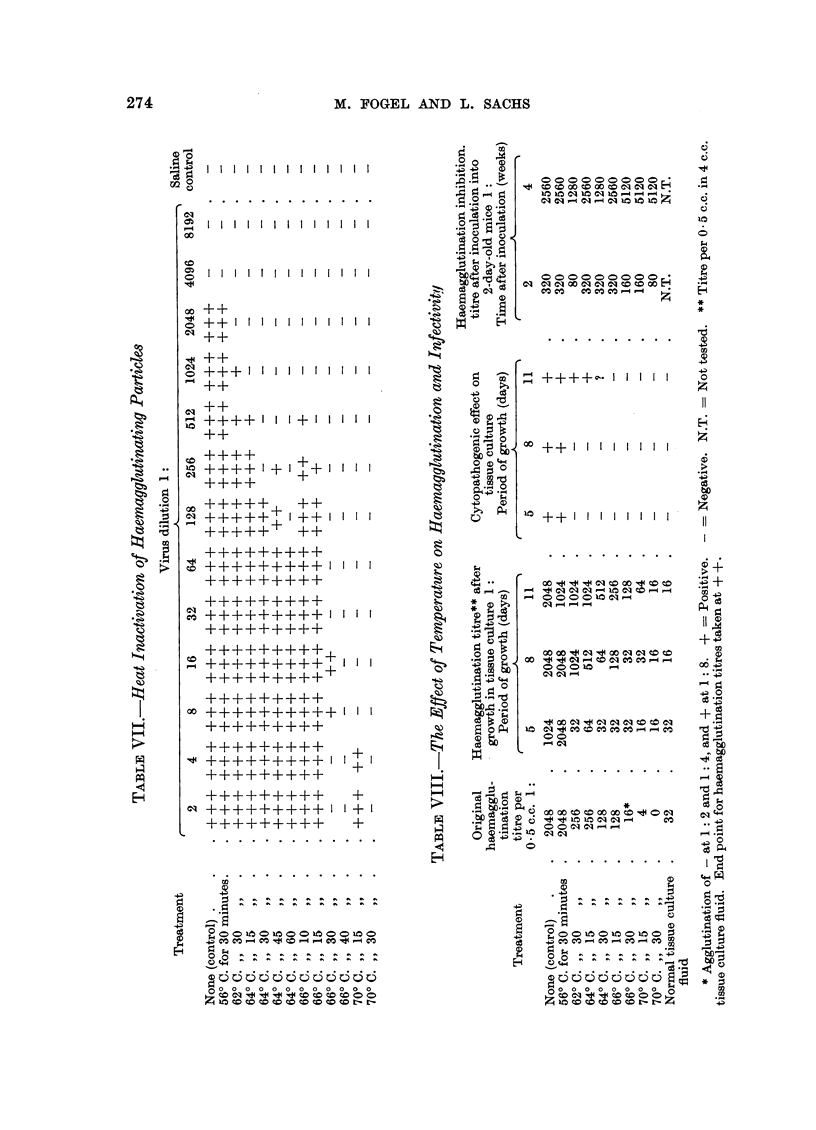

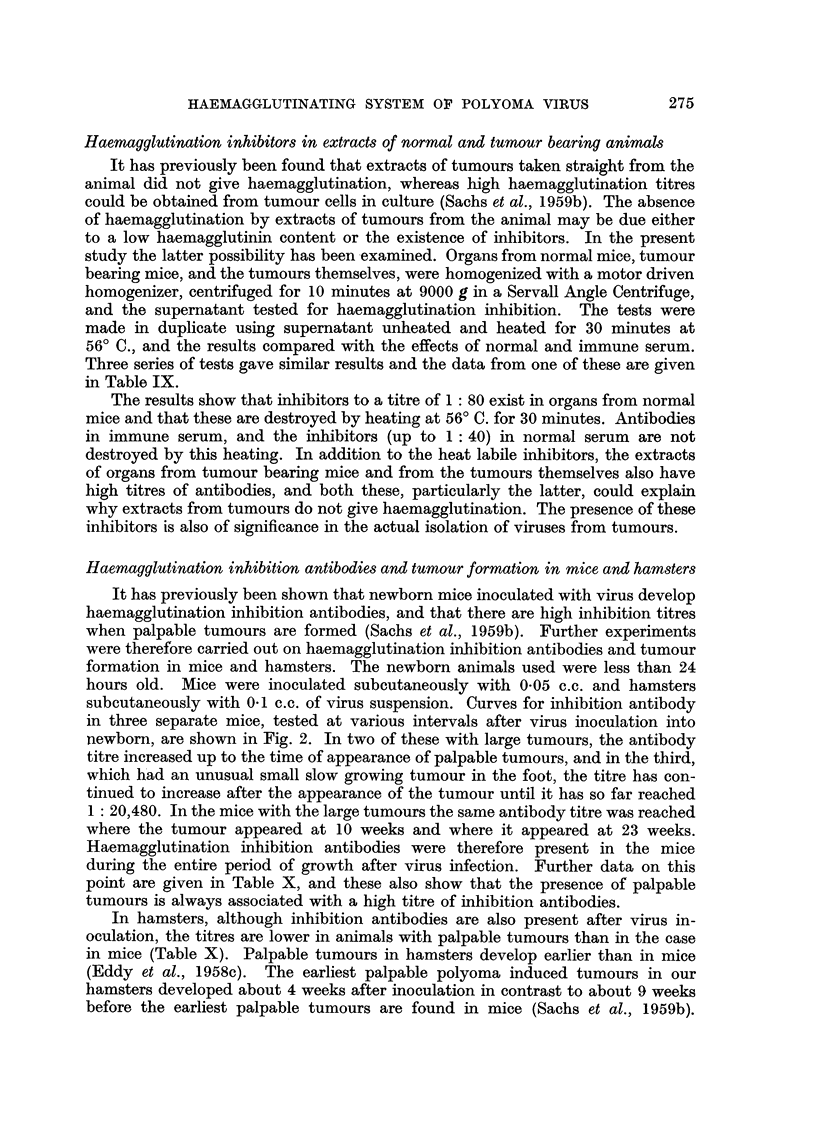

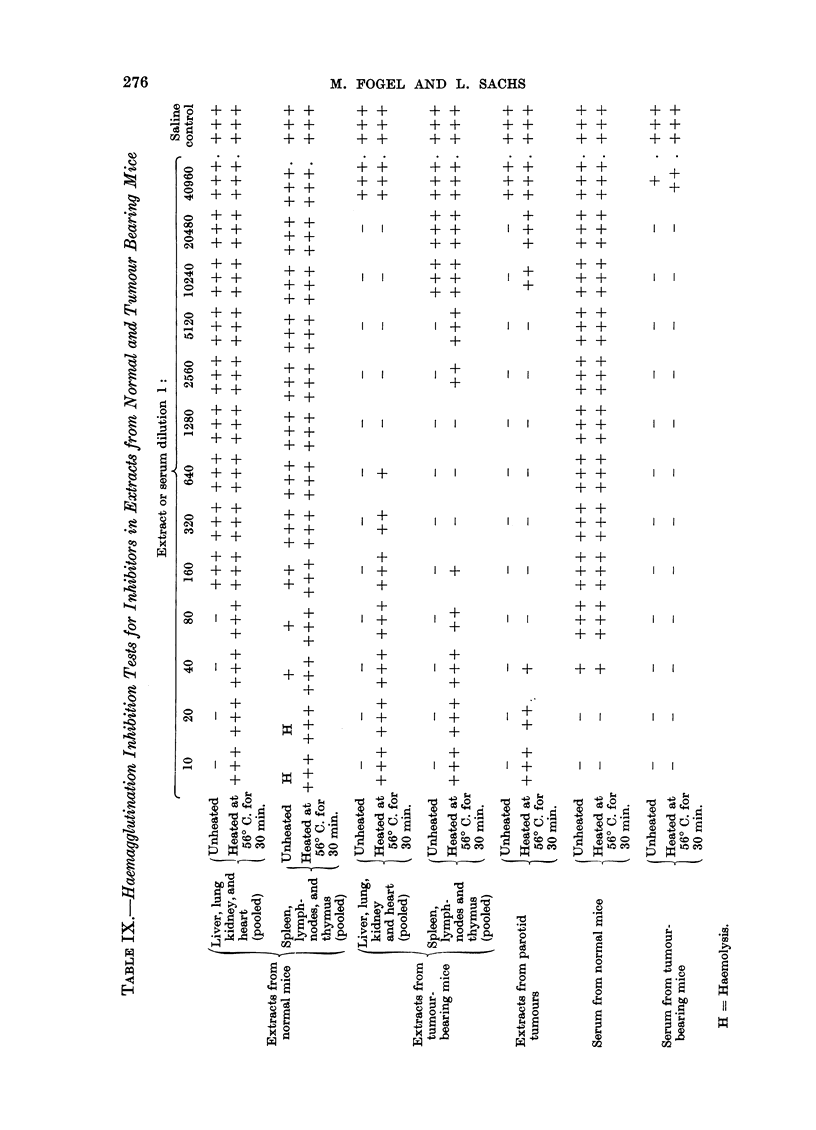

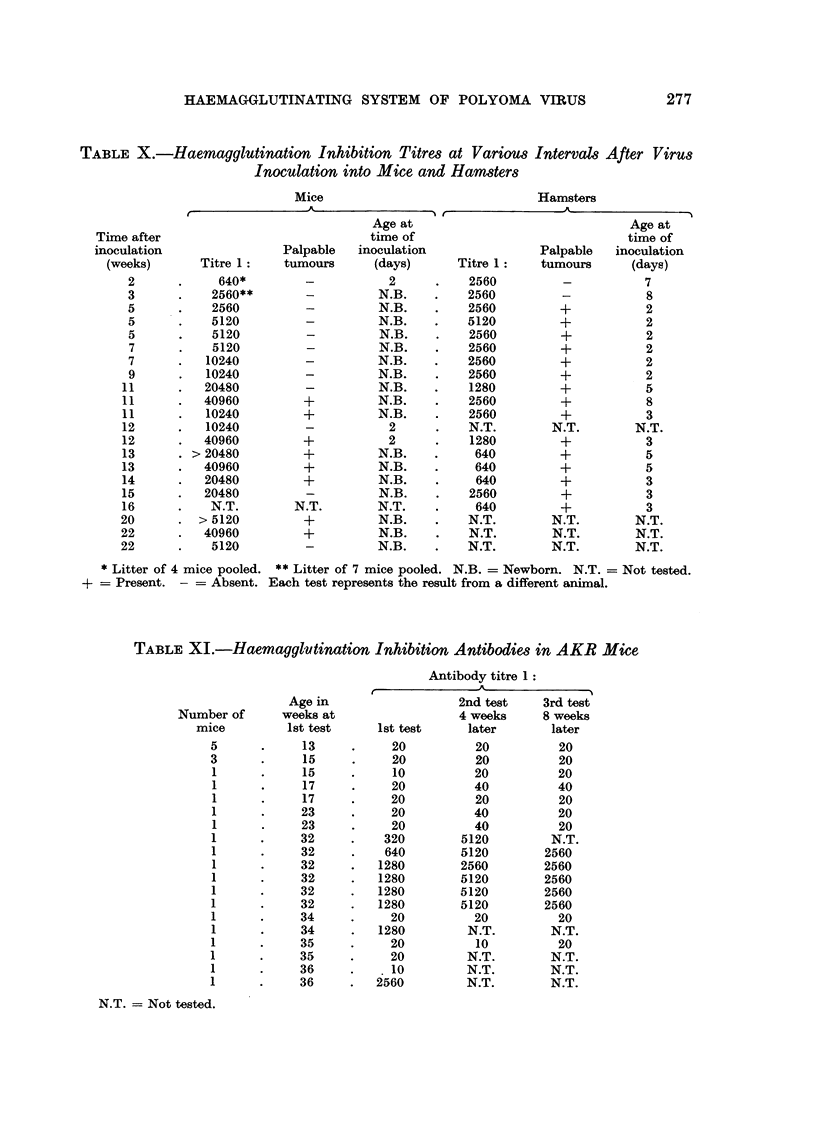

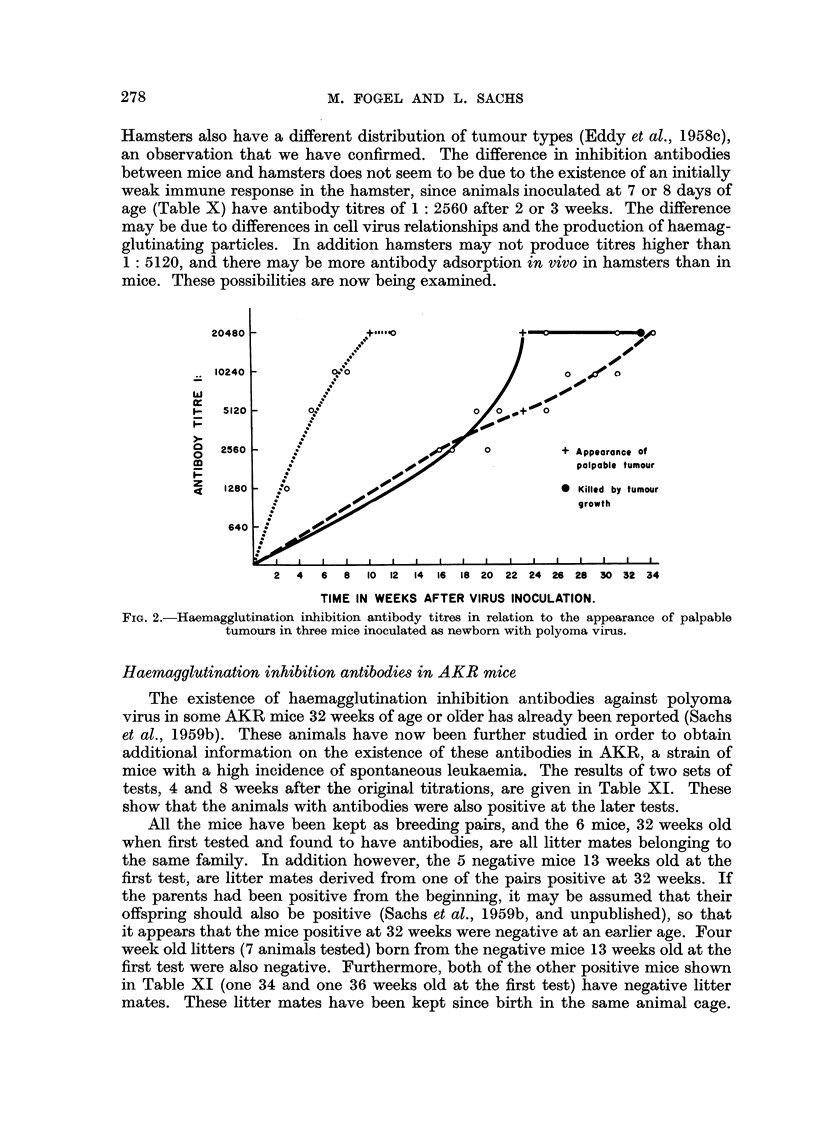

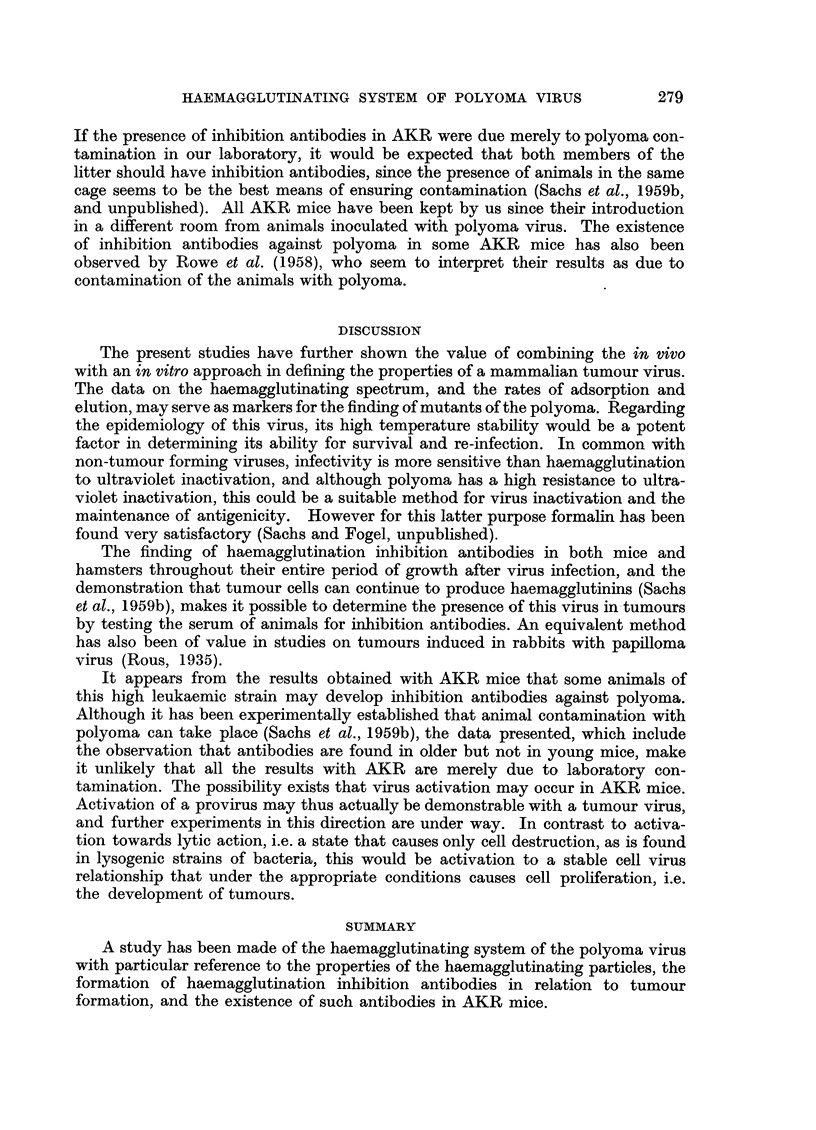

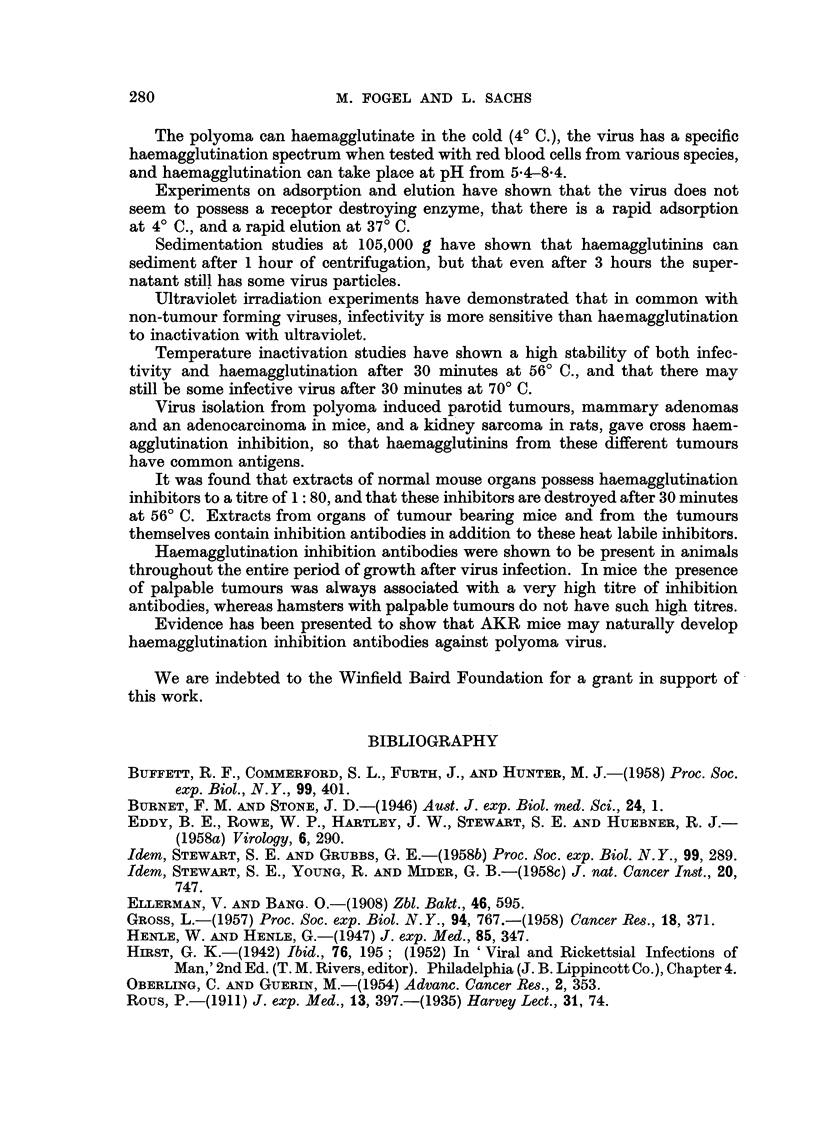

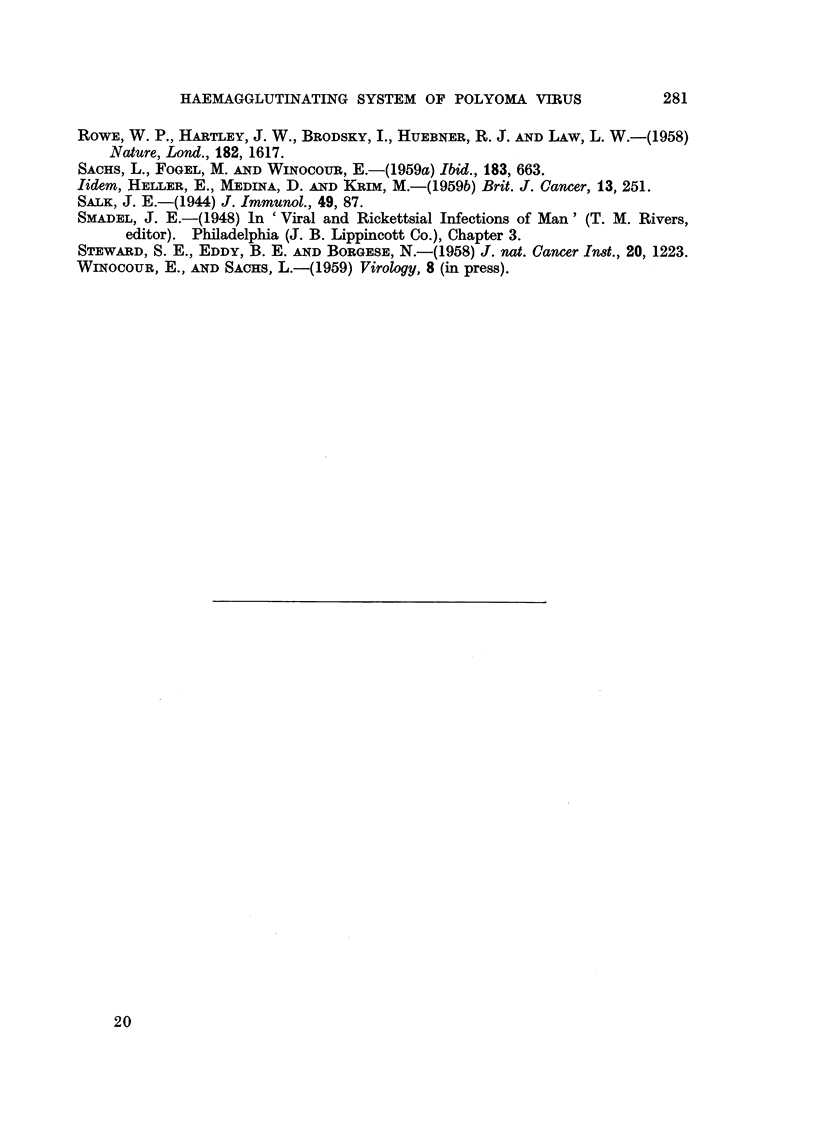

